# Subclinical Reactivation of Cytomegalovirus Drives CD4^+^CD28^null^ T-Cell Expansion and Impaired Immune Response to Pneumococcal Vaccination in Antineutrophil Cytoplasmic Antibody–Associated Vasculitis

**DOI:** 10.1093/infdis/jiy493

**Published:** 2018-08-09

**Authors:** Dimitrios Chanouzas, Michael Sagmeister, Sian Faustini, Peter Nightingale, Alex Richter, Charles J Ferro, Matthew David Morgan, Paul Moss, Lorraine Harper

**Affiliations:** 1 Institute of Inflammation and Ageing, College of Medical and Dental Sciences, University of Birmingham, United Kingdom; 2 Department of Nephrology, University Hospitals Birmingham National Health Service Foundation Trust, United Kingdom; 3 Institute of Immunology and Immunotherapy, College of Medical and Dental Sciences, University of Birmingham, United Kingdom; 4 Institute of Translational Medicine Birmingham, United Kingdom; 5 Institute of Clinical Sciences, College of Medical and Dental Sciences, University of Birmingham, United Kingdom

**Keywords:** cytomegalovirus, CD4^+^CD28^null^, valacyclovir, pneumococcal vaccination, clinical trial

## Abstract

**Background:**

Infection is the leading cause of death in antineutrophil cytoplasmic antibody–associated vasculitis (AAV). Expansion of CD4^+^CD28^null^ T cells is associated with increased risk of infection and mortality, but is only present in cytomegalovirus (CMV)–seropositive individuals. We hypothesized that subclinical CMV reactivation drives CD4^+^CD28^null^ T-cell expansion, that this is associated with impaired immune response to heterologous antigens, and that antiviral therapy may ameliorate this.

**Methods:**

In a proof-of-concept open-label clinical trial, 38 CMV-seropositive AAV patients were randomized to receive valacyclovir for 6 months or no intervention. CMV reactivation was measured monthly in plasma and urine. CD4^+^CD28^null^ T cells were enumerated at baseline and at 6 months. At 6 months, 36 patients were vaccinated with a 13-valent pneumococcal vaccine. Serotype-specific immunoglobulin G was assayed before and 4 weeks postvaccination to calculate the antibody response ratio.

**Results:**

Valacyclovir treatment suppressed subclinical CMV reactivation and reduced CD4^+^CD28^null^ T-cell proportion. CD4^+^CD28^null^ T-cell reduction correlated with improved vaccine response, whereas CMV reactivation associated with reduced response to vaccination. Furthermore, expansion of CD4^+^CD28^null^ T cells was associated with a reduction in the functional capacity of the CD4 compartment.

**Conclusions:**

Suppression of CMV may improve the immune response to a T-cell–dependent pneumococcal vaccination in patients with AAV, thus offering potential clinical benefit.

**Clinical Trials Registration:**

NCT01633476.

## Teaser

Infection is a leading cause of death in antineutrophil cytoplasmic antibody–associated vasculitis. Here we show that suppression of subclinical reactivation of cytomegalovirus limits CD4^+^CD28^null^ T-cell expansion and that this is associated with improved immune response to a T-cell–dependent pneumococcal vaccine challenge.

The antineutrophil cytoplasmic antibody (ANCA)–associated vasculitides (AAVs) are autoimmune, inflammatory conditions characterized by necrotizing inflammation affecting small to medium blood vessels, leading to end organ damage [1]. The introduction of successful immunosuppressive regimens for inducing disease remission has transformed their management. However, patients with AAV continue to experience significant excess morbidity and mortality, with infection being the leading cause of death [2]. Respiratory infections are very common among AAV patients and although pneumococcal vaccination is indicated, vaccine responses are suboptimal [3]. In addition, impaired vaccine response is a marker of poor immunologic function and is independently associated with increased all-cause mortality [3].

We have previously demonstrated in patients with AAV that the expansion of a subset of CD4 T cells that have lost expression of the costimulatory molecule CD28 (CD4^+^CD28^null^ T cells) is independently associated with risk of infection and all-cause mortality [4]. CD4^+^CD28^null^ T cells are proinflammatory and expand under inflammatory conditions [4–6]. Furthermore, this expansion is associated with a reduction in naive CD4 T cells [4], suggesting a consequent diminished ability of the immune system to respond to new antigenic challenges. Loss of CD28 suggests repeated exposure to a persistent antigen [7] and we, and others, have demonstrated that significant expansion of CD4^+^CD28^null^ T cells occurs exclusively in cytomegalovirus (CMV)–seropositive individuals [4, 8–11]. CMV infection itself has been linked to impaired immune response to heterologous antigens [12–15] and has been implicated as a driver of the observed age-associated decline in immune function in the general population [12, 16–19].

We hypothesized that subclinical asymptomatic reactivation of CMV drives expansion of CD4^+^CD28^null^ T cells in AAV and that this is associated with impaired immune response to heterologous antigens. Furthermore, we postulated that antiviral therapy might act to ameliorate this effect, offering potential clinical benefit. To investigate this, we conducted a proof-of-concept open-label clinical trial of valacyclovir therapy in CMV-seropositive AAV patients in remission and vaccinated patients at the end of treatment with a T-cell–dependent 13-valent pneumococcal conjugate vaccine (PCV13).

## MATERIALS AND METHODS

Thirty-eight CMV-seropositive patients with AAV in stable remission were recruited from the vasculitis clinic at University Hospitals Birmingham (UHB) National Health Service Foundation Trust (Birmingham, United Kingdom) and participated in a single-center, proof-of-concept, open-label, randomized clinical trial of oral valacyclovir vs standard care (no anti-CMV treatment) (ClinicalTrials.gov identifier NCT01633476). The study was approved by the Research Ethics Committee of Yorkshire and The Humber, United Kingdom. The trial study protocol has been published previously [20]. Written informed consent was obtained from all participants.

### Clinical Trial

The trial was designed to determine the safety and efficacy of valacyclovir-induced CMV suppression in AAV and to investigate whether prevention of subclinical CMV reactivation limits expansion of CD4^+^CD28^null^ T cells.

Patients were eligible for inclusion if they had a documented diagnosis of AAV, were in stable remission for at least 6 months, on maintenance immunosuppression with no more than 1 agent in addition to prednisolone (prednisolone dose ≤5 mg), and were CMV seropositive (anti-CMV immunoglobulin G [IgG] detected in blood). Exclusion criteria were estimated glomerular filtration rate <15 mL/minute/1.73m^2^, B-cell–depleting therapy within 12 months or T-cell–depleting therapy within 6 months, presence of other chronic infection (human immunodeficiency virus, hepatitis B virus, hepatitis C virus, tuberculosis), or treatment with anti-CMV therapies within the previous month.

Between 1 August 2013 and 28 February 2016, eligible patients were randomized (1:1) to receive 6 months of oral valacyclovir (2 g 4 times a day; dose adjusted according to creatinine clearance), or no additional therapy, and followed up for an additional 6 months. Block randomization by CD4^+^CD28^null^ T-cell percentage (<40% or ≥40%) was used (Primary Care Clinical Research and Trials Unit, Birmingham, United Kingdom). The randomization used mixed blocks of random size not known to the investigators. Although patients and investigators were not blinded, laboratory staff undertaking the CMV quantitative polymerase chain reaction (qPCR) assay that informed the primary outcome were blinded to treatment allocation. An interim safety analysis was undertaken after 10 patients completed treatment.

The clinical trial primary outcome was time to first CMV reactivation in blood or urine (defined as ≥200 viral copies/mL) within 6 months of randomization. Reactivation episodes detected at baseline prior to treatment commencement were not included in this analysis. Secondary outcomes were change in CD4^+^CD28^null^ T-cell proportion from baseline to end of treatment, change in soluble markers of inflammation from baseline to end of treatment, and number of adverse events.

### Vaccination and Immunological Measure of Response

At 6 months, 36 of 38 AAV patients (18 treated/18 controls) were vaccinated with a T-cell–dependent PCV13 (0.5 mL; Prevnar, Pfizer) as per clinical recommendations [21]. Participants had not received pneumococcal vaccination in the preceding 5 years. Blood was drawn prior to vaccination and 4 weeks postvaccination to determine serotype-specific anti-IgG titer for 12 pneumococcal serotypes contained in PCV13 (serotypes 1, 3, 4, 5, 6b, 7f, 9v, 14, 18c, 19a, 19f, 23f) using a multiplex assay quality assured externally by the United Kingdom National External Quality Assessment Service [22]. Serum was separated from blood by centrifugation and cryopreserved at –80°C until analysis. Because a protective level of serum antibody has not been strictly defined and may differ among serotypes, we used the antibody response ratio (ARR) as a measure of immune response to vaccination as previously employed by others [23]. ARR was calculated (antibody titer at 4 weeks postvaccination / antibody titer prior to vaccination), and mean ARR (sum of ARR in all serotypes assessed / number of serotypes) was utilized as a single measure of immune response.

### Blood Collection

Plasma was isolated by centrifugation and cryopreserved at –80°C. Peripheral blood mononuclear cells (PBMCs) were isolated from heparinized blood by density gradient centrifugation and used immediately in stimulation experiments with staphylococcal enterotoxin B (SEB) to identify cytokine-producing T cells.

Cells for flow cytometry experiments were acquired on a BD LSRII Flow Cytometer and analyzed using FACSDiva version 8.0 software (BD). Monoclonal antibodies used for flow cytometry experiments, and gating strategies are shown in Supplementary Table 1 and Supplementary Figures 1 and 2).

### Enumeration of Peripheral Blood CD4^+^CD28^null^ T Cells

Whole blood was stained with anti-CD3, anti-CD4, and anti-CD28 monoclonal antibodies to determine CD4^+^CD28^null^ T-cell percentage. Quality control was achieved by using a positive control (Cytofix CD4 Positive Control, Cytomark) with a validated acceptance range for CD3^+^CD4^+^ percentage, and a fluorescence-minus-1 control to aid CD28 gating. Absolute counts were determined by adding counting beads (CytoCount, Dako) prior to acquisition as previously described [20].

### PBMC Stimulation

To identify cytokine-producing T cells following activation, 5 × 10^5^–1 × 10^6^ PBMCs were resuspended in supplemented medium (RPMI, 10% sterile filtered heat-inactivated fetal calf serum [Sigma-Aldrich], 1% penicillin/streptomycin [Thermo Fisher Scientific]) overnight for 16 hours at 37°C, 5% carbon dioxide, in the presence of monensin (2 μM). Cells were co-cultured with saturating amounts of anti-CD154 monoclonal antibody conjugated to phycoerythrin (Ebioscience) and stimulated with SEB (0.2 μg/mL; Sigma-Aldrich) to assess CD4 T-cell functional capacity. Unstimulated cells served as controls. Following overnight incubation, cells were stained with fixable viability dye eFluor-506 (Ebioscience), then costained with saturating amounts of anti-CD3, anti-CD4, and anti-CD28 monoclonal antibodies for 30 minutes at 4°C, washed with flow cytometry buffer, fixed, and permeabilized using an intracellular flow cytometry staining kit (Ebioscience) according to the manufacturer’s instructions. Cells were then stained with anti–interferon gamma (IFN-γ), anti–tumor necrosis factor alpha (TNF-α), and anti–interleukin 2 (IL-2) monoclonal antibodies. The ability of IFN-γ–positive SEB-responsive cells to coproduce TNF-α, IL-2, and CD154 was evaluated as a measure of CD4 T-cell functional capacity.

### CMV Viral Load Quantification

Plasma and urine samples from clinical trial patients were analyzed at monthly intervals until the end of the study (month 12) by the UHB Virology Laboratory using a clinically validated CMV DNA qPCR assay (RealTime CMV, Abbott). The lower limit of quantification was 200 viral copies/mL.

### Measurement of Soluble Markers of Inflammation

Soluble markers of inflammation (IL-2, TNF-α, IFN-γ, interleukin 10, interleukin 17A, interleukin 6, and high-sensitivity C-reactive protein [hs-CRP]) were measured in plasma by Luminex array (ProcartaPlex, Ebioscience) according to the manufacturer’s instructions and read on a Bio-Rad Luminex 200 instrument (Bio-Rad, Hercules).

### Anti-CMV IgG Titer Determination

Plasma anti-CMV IgG titer was assayed using an enzyme-linked immunosorbent assay as previously described [24].

### Statistical Analysis

Correlations were assessed with Spearman rank correlation. All clinical trial analyses were performed on an intention-to-treat principle. Time to first reactivation was compared between control and treatment groups by constructing Kaplan–Meier plots; Gehan–Breslow–Wilcoxon test was used to report hazard ratio and 95% confidence interval (CI). Secondary and exploratory outcome data were analyzed using paired *t* tests. As the ratios of paired values for CD4^+^CD28^null^ T cells and plasma markers of inflammation were expected to be more consistent than the differences, paired ratio *t* tests were used. Change in anti-CMV IgG titer over the study period was analyzed using a post hoc test for linear trend to evaluate change over time (expressed as slope). Between-group comparisons were performed using the Mann–Whitney *U* or χ^2^ tests with Fisher exact test where appropriate. Analyses were undertaken using SPSS Statistics version 21 (IBM Corporation) and GraphPad Prism version 5 software and were 2-tailed; *P* value <.05 was considered significant.

## RESULTS

Baseline characteristics of study participants are shown in Table 1.

**Table 1.** Participant Baseline Characteristics

Characteristic	Treatment Arm (n = 19)	Control Arm (n = 19)	*P* Value^a^
Age, y, median (IQR)	69.0 (60.9–75.0)	67.0 (64.1–75.7)	.530
Sex, male:female	12:7	13:6	.732
ANCA specificity, PR3:MPO	12:6^b^	15:4	.476
AAV disease chronicity, y, median (IQR)	7.3 (2.8–11.5)	5.8 (3.2–11.4)	.930
Renal function (eGFR), mL/min/1.73m^2^, mean (SD)	53 (22)	59 (18)	.339
uACR, mg/mmol, median (IQR)	1.8 (0.8–6.8)	4.4 (1.8–7.5)	.148
Steroids, No. (%)	13 (68.4)	15 (78.9)	.714
MMF, No. (%)	5 (26.3)	5 (26.3)	1.000
Azathioprine, No. (%)	6 (31.6)	8 (42.1)	.501
No current immunosuppression, No. (%)	2 (10.5)	1 (5.3)	1.000
CD4^+^CD28^null^ % at prerandomization visit, median (IQR)	10.9 (2.5–15.8)	19.1 (7.5–25.1)	.102

Immunosuppressive treatment refers to number and percentage of patients on the respective immunosuppressive agent at the time of study entry. Maintenance immunosuppression dosage: prednisolone 5 mg once a day; MMF 250–500 mg twice a day; azathioprine 1.5–2.0 mg/kg/day.

Abbreviations: AAV, antineutrophil cytoplasmic antibody–associated vasculitis; ANCA, antineutrophil cytoplasmic antibody; eGFR, estimated glomerular filtration rate; IQR, interquartile range; MMF, mycophenolate mofetil; MPO, myeloperoxidase; PR3, proteinase 3; SD, standard deviation; uACR, urine albumin-to-creatinine ratio.

^a^Comparison between treatment and control arms.

^b^One patient was ANCA negative.

### Subclinical CMV Reactivation Drives the Expansion of CD4^+^CD28^null^ T-Cells and This Is Limited by Antiviral Therapy

**Figure 1.**
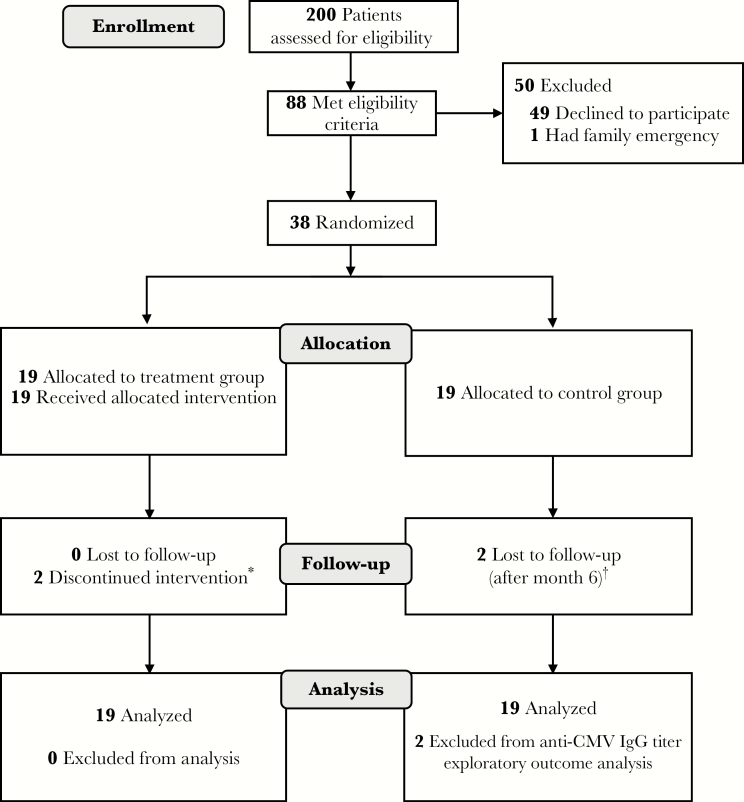
Clinical trial flowchart. ^*^One patient electively stopped taking the study drug within 1 month of commencement. One patient developed an episode of acute kidney injury that led to discontinuation of valacyclovir within 1 month. Both patients completed subsequent trial visits fully, although the study drug was not restarted. ^†^Two patients in the control group declined to attend visits following the initial 6-month period. Abbreviations: CMV, cytomegalovirus; IgG, immunoglobulin G.

To determine whether subclinical CMV reactivation drives the expansion of CD4^+^CD28^null^ T cells and if this can be reversed with antiviral therapy, 38 CMV-seropositive AAV patients in stable remission were randomized to 6 months of oral valacyclovir (n = 19) or no additional treatment (n = 19) (Table 1 and Figure 1). Valacyclovir was well tolerated. Gastrointestinal adverse events were more common in treated patients as expected (Supplementary Table 2), but were mild and transient in nature. Valacyclovir treatment was discontinued in 1 patient who developed an episode of acute kidney injury, but renal function returned to baseline upon cessation of the drug. There were no AAV disease relapses during the study.

**Figure 2.**
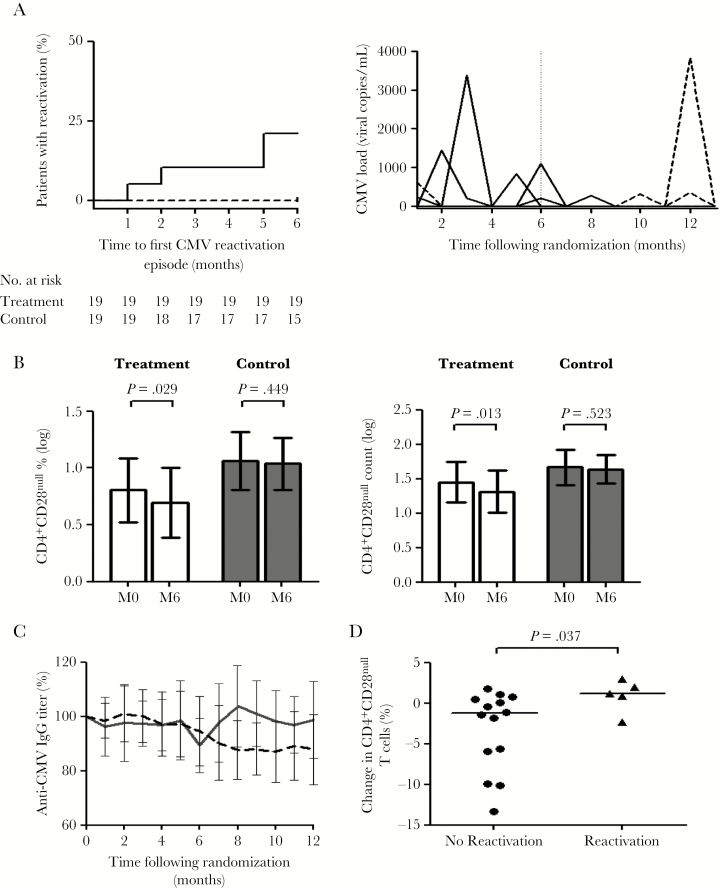
Subclinical cytomegalovirus (CMV) reactivation drives the expansion of CD4^+^CD28^null^ T cells, and antiviral therapy limits this expansion. *A*, Time to first CMV reactivation in treatment (n = 19, dashed) vs control (n = 19, solid) groups (hazard ratio, 8.2; 95% confidence interval [CI], 1.1–59.1; *P* = .037) and reactivation episodes in treated (dashed) and control (solid) patients during the course of the study. On the second plot, each line represents a single patient; the end of the treatment period is indicated by a dashed vertical line at month 6. *B*, There was a significant reduction in CD4^+^CD28^null^ T-cell percentage and absolute count from baseline (M0) to end of treatment (M6) in treated patients. There was no change in controls. Bars show mean with 95% CI. *C*, Proportionate change in anti-CMV immunoglobulin G (IgG) titer during the course of the study. There was a significant reduction in anti-CMV IgG titer in treated patients (dashed line; slope –1.305; *P* < .001). There was no significant change in controls (solid line; slope 0.218; *P* = .521). *D*, Control patients (n = 19) with CMV reactivation had an increase in CD4^+^CD28^null^ T cells during the course of the study compared to patients with no reactivation. Bars show medians. Abbreviations: CMV, cytomegalovirus; IgG, immunoglobulin G; M0, baseline; M6, month 6.

Valacyclovir treatment completely suppressed CMV reactivation. During the 6-month treatment period, viral reactivation was observed in 4 patients in the control group (21.1%) but not observed in the treatment group (hazard ratio, 8.2 [95% CI, 1.1–59.1; *P* = .037; Figure 2A). One episode of reactivation was detected in a patient from the control group and 1 episode in a patient from the treatment group at the baseline visit prior to commencement of valacyclovir (Figure 2A). These 2 episodes were not included in the primary outcome analysis. Following the end of the treatment period, CMV reactivation was detected in 3 patients within the treatment group (Figure 2A). All CMV reactivation episodes were asymptomatic and only detected in urine.

At the end of the treatment period, there was a –23% (95% CI, –38.9% to –3.0%; *P* = .029; paired ratio *t* test) reduction in the percentage of CD4^+^CD28^null^ T cells in valacyclovir-treated patients compared with baseline. No significant change in the percentage of CD4^+^CD28^null^ T cells was seen in the control group (–5.4% [95% CI, –18.6% to 11.0%]; *P* = .449; paired ratio *t* test; Figure 2B). Analysis with absolute CD4^+^CD28^null^ T-cell counts revealed a reduction in the absolute CD4^+^CD28^null^ T-cell count in treated patients (–27.0% [95% CI, –42.6% to –7.1%]; *P* = .013) and, again, no change in the control group (–6.6% [95% CI, –25.0% to 16.3%]; *P* = .523). This indicates that the CD4^+^CD28^null^ T-cell percentage reduction seen in valacyclovir-treated patients reflected a true reduction in CD4^+^CD28^null^ T cells rather than changes in other CD4 lymphocyte subsets.

A reduction in the plasma levels of IL-2 and IFN-γ, cytokines known to be produced by CD4^+^CD28^null^ T cells, occurred only in treated patients (Supplementary Table 2). In addition, there was a delayed but persistent reduction in the anti-CMV IgG titer in valacyclovir-treated patients (slope –1.31; *P* < .001) but not in controls (slope 0.218; *P* = .521) (Figure 2C).

To confirm the impact of subclinical CMV reactivation on the expansion of CD4^+^CD28^null^ T cells, a post hoc analysis was carried out in control patients to investigate the relationship between change in the percentage of CD4^+^CD28^null^ T cells and episodes of viral reactivation. Control patients who had at least 1 episode of CMV reactivation had an increase in the percentage of CD4^+^CD28^null^ T cells compared to those who did not reactivate (1.2% [interquartile range {IQR}, –0.7% to 2.5%] vs –1.3% [IQR, –6.9 to 0.6]; *P* = .037; Figure 2D). Furthermore, the increase in CD4^+^CD28^null^ T-cell percentage correlated with the number of reactivation episodes (ρ = 0.523; *P* = .022).

### Subclinical CMV Reactivation and Consequent Expansion of CD4^+^CD28^null^ T-Cells Is Associated With Impaired Immune Response to Pneumococcal Vaccination

To determine whether subclinical reactivation of CMV and the consequent expansion of the CD4^+^CD28^null^ T-cell subset is linked to reduced response to heterologous antigens, 36 of the 38 trial patients (18 treated, 18 controls) were vaccinated with a T-cell–dependent PCV13 at the end of the valacyclovir treatment period. Analysis of prevaccination and 4-week postvaccination serotype-specific IgG titers revealed a statistically significant increase in all pneumococcal serotypes across the entire patient group, with the exception of serotype 14 (Supplementary Figure 3).

**Figure 3.**
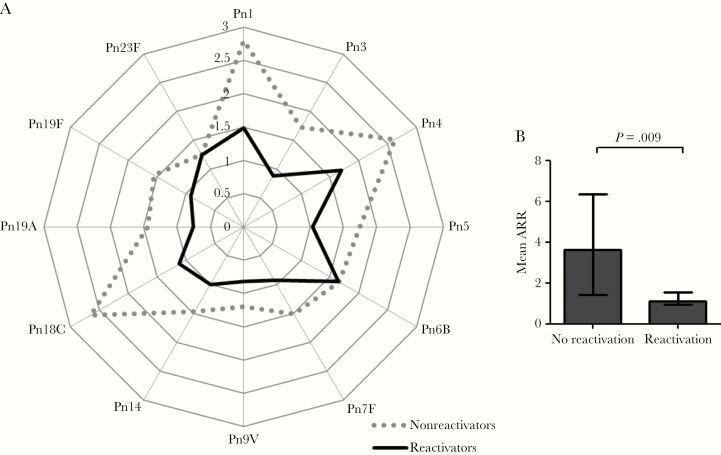
Subclinical reactivation of cytomegalovirus (CMV) is associated with impaired immune response to pneumococcal vaccination. *A*, Spider graph showing median antibody response ratio (ARR) for each individual pneumococcal serotype for patients with subclinical CMV reactivation during the 6 months prior to administration of 13-valent pneumococcal conjugate vaccine (PCV13) (n = 5, solid line) and those without (n = 31, dotted line). *B*, Patients with subclinical CMV reactivation (n = 5) had a lower mean ARR to PCV13 compared to those without (n = 31). Abbreviations: ARR, antibody response ratio; Pn, pneumococcal serotype.

Patients with evidence of subclinical CMV reactivation during the 6 months preceding vaccination had a significantly suppressed immune response to PCV13 (ARR) across all pneumococcal serotypes analyzed, compared to individuals with no CMV reactivation (Figure 3A). Similarly, individuals with subclinical CMV reactivation exhibited a markedly lower mean ARR compared to those with no reactivation (1.1 [IQR, 1.0–1.6] vs 3.6 [IQR, 1.4–6.4]; *P* = .009; Figure 3B).

**Figure 4.**
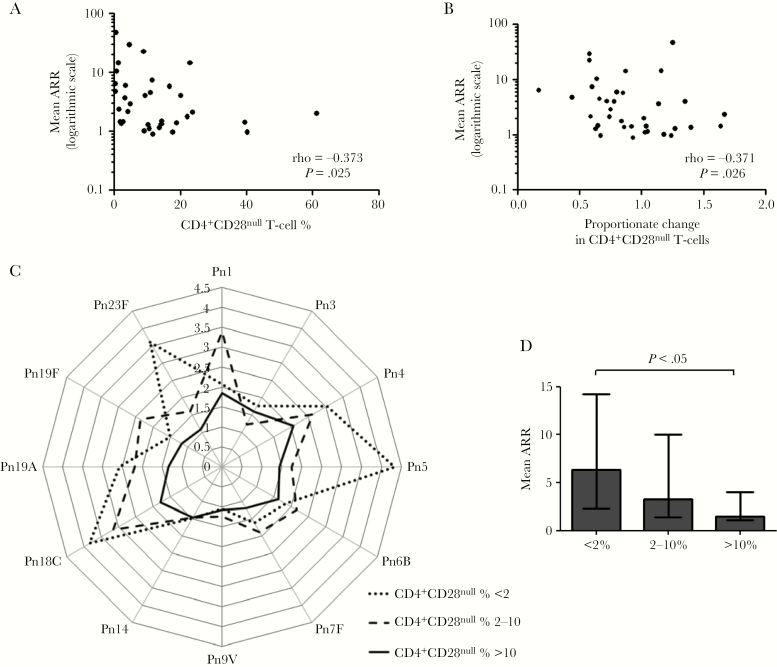
Expansion of CD4^+^CD28^null^ T cells is associated with impaired immune response to pneumococcal vaccination. *A*, Correlation between prevaccination CD4^+^CD28^null^ T-cell percentage (at end of valacyclovir treatment period) and mean antibody response ratio (ARR) to 13-valent pneumococcal conjugate vaccine (PCV13) (n = 36). *B*, Correlation between proportionate change in CD4^+^CD28^null^ T-cell percentage from baseline to end of valacyclovir treatment period (month 6 CD4^+^CD28^null^ T-cell % / baseline CD4^+^CD28^null^ T-cell %) and mean ARR to PCV13 (n = 36). *C*, Spider graph showing median ARR for each individual pneumococcal serotype for patients with prevaccination CD4^+^CD28^null^ T cells <2% (n = 7; dotted line), 2%–10% (n = 10; dashed line), and >10% (n = 19; solid line). *D*, Mean ARR across all serotypes measured for patients with prevaccination CD4^+^CD28^null^ T-cell percentage <2%, 2%–10%, and >10%, indicating patients with a low, moderate, and high impact, respectively, of CMV on the immune system, exhibiting a graded immune response to PCV13 vaccination across these 3 categories. Bars represent the median, and error bars represent the interquartile range. Abbreviations: ARR, antibody response ratio; Pn, pneumococcal serotype.

At the end of valacyclovir treatment, the size of the CD4^+^CD28^null^ T-cell expansion immediately prior to vaccination with PCV13 was inversely correlated with the antibody response (mean ARR, ρ = –0.373; *P* = .025; Figure 4A). Furthermore, reduction in CD4^+^CD28^null^ T cells was associated with better immune response to PCV13; change in CD4^+^CD28^null^ T-cell percentage from baseline to end of treatment was inversely correlated with mean ARR (ρ = –0.371; *P* = .026) (Figure 4B). Mean ARR was not associated with patient age, sex, renal function, hs-CRP, immunosuppression, or the humoral immune response to CMV (anti-CMV IgG titer) (data not shown).

To further investigate the relationship between the size of the CD4^+^CD28^null^ T-cell expansion and PCV13 response, we categorized patients according to prevaccination CD4^+^CD28^null^ T-cell percentage (<2%, 2%–10%, >10%). The cutoff of <2% identified patients with a low impact of CMV on the immune system and was based on previous observations that accumulation of CD4^+^CD28^null^ T cells >2% is not seen among CMV-seronegative individuals [4, 25]. All patients who had at least 1 episode of subclinical CMV reactivation during the treatment period had a CD4^+^CD28^null^ T-cell percentage >10%. A CD4^+^CD28^null^ T-cell percentage >10% therefore identified patients with a high impact of CMV on the immune system, whereas a CD4^+^CD28^null^ T-cell percentage between 2% and 10% identified those with a moderate impact. We observed a graded immune response in individual serotype ARR (Figure 4C), as well as mean ARR (Figure 4D), across the 3 categories. Individuals with large expansions of CD4^+^CD28^null^ T cells (>10%) had the lowest mean ARR, whereas those with reduced CD4^+^CD28^null^ T-cell expansions (<2%) exhibited the highest mean ARR (Figure 4D).

### Expansion of the CD4^+^CD28^null^ T-Cell Subset Is Associated With Reduced Functional Capacity of the CD4 Compartment

Given that PCV13 is a T-cell–dependent pneumococcal vaccine, we next sought to determine whether expansion of the CD4^+^CD28^null^ T-cell subset is associated with reduced functional capacity of the CD4 compartment. To address this, PBMCs were stimulated with a superantigen (SEB) immediately prior to PCV13 vaccination. SEB-responsive CD4 T cells were identified by IFN-γ expression. Functional capacity was then evaluated by assessing for coexpression of the activation marker CD154 and cytokines TNF-α and IL-2 on SEB-responsive CD4 T cells.

**Figure 5.**
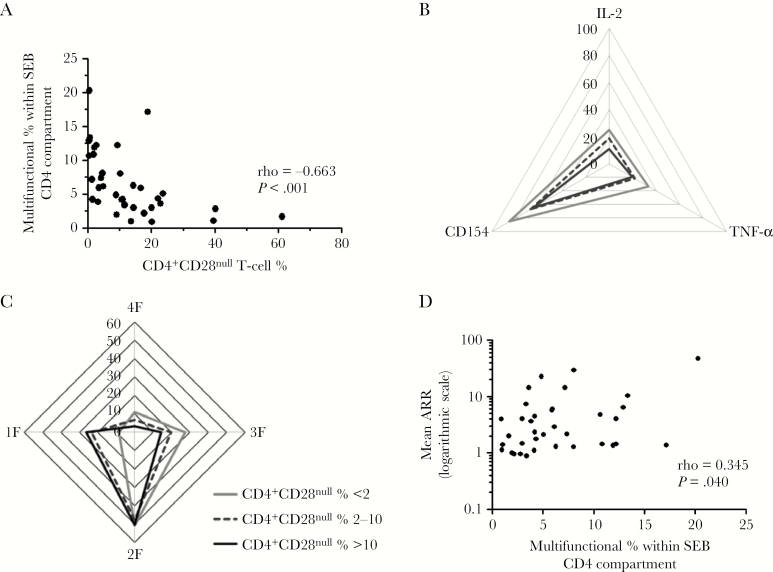
Expansion of CD4^+^CD28^null^ T cells is associated with reduced functional capacity of the CD4 compartment. *A*, Correlation between CD4^+^CD28^null^ T-cell percentage and proportion of multifunctional (CD154^+^tumor necrosis factor alpha [TNF-α]^+^interleukin 2 [IL-2]^+^) cells within staphylococcal enterotoxin B (SEB)–responsive interferon gamma (IFN-γ^+^) CD4 T cells. *B*, Spider graph showing median percentage of cells with CD154, TNF-α, or IL-2 coexpression within SEB-responsive CD4 T cells in patients with small (<2%, gray line), moderate (2%–10%, dashed line), and large (>10%, solid black line) CD4^+^CD28^null^ T-cell expansions. *C*, Spider graph showing median percentage of cells with IFN-γ expression only (1F), IFN-γ and one of CD154, TNF-α, or IL-2 coexpression (2F; double functional), IFN-γ and 2 of CD154, TNF-α, or IL-2 coexpression (3F; triple functional), or multifunctional (4F) capacity within SEB-responsive CD4 T cells for patients with small (<2%, gray line), moderate (2%–10%, dashed line), and large (>10%, solid black line) CD4^+^CD28^null^ T-cell expansions. *D*, Correlation between proportion of multifunctional (4F) cells within the SEB-responsive CD4 compartment and mean antibody response ratio to the 13-valent pneumococcal conjugate vaccine. Abbreviations: IL-2, interleukin 2; SEB, staphylococcal enterotoxin B; TNF-α, tumor necrosis factor alpha.

Increasing CD4^+^CD28^null^ T-cell percentage was strongly correlated with a reduction in the proportion of multifunctional (CD154^+^TNF-α^+^IL2^+^) SEB-responsive CD4^+^ T cells (ρ = –0.663; *P* < .001; Figure 5A). In keeping with these findings, SEB-responsive CD4^+^ T cells from individuals with large CD4^+^CD28^null^ T-cell expansions (>10%) were less likely to coexpress either TNF-α, IL-2, or CD154, compared to cells from individuals with moderately sized (2%–10%) or small (<2%) expansions, in a graded fashion (Figure 5B). The same relationship was observed when multifunctional capacity was assessed. SEB-responsive CD4^+^ T cells from patients with large CD4^+^CD28^null^ T-cell expansions were more likely to be single or double functioning and less likely to be triple or multifunctioning compared to cells from individuals with moderately sized or small expansions (Figure 5C).

Taken together, these data indicate that increasing CD4^+^CD28^null^ T-cell expansion is associated with a progressive decline in functional capacity within the CD4 compartment as a whole. Moreover, the proportion of multifunctional (CD154^+^TNF-α^+^IL2^+^) cells within the SEB-responsive CD4 compartment was positively correlated with the antibody response to PCV13 (ρ = 0.345; *P* = .040; Figure 5D), suggesting that reduction in functional capacity of the CD4 compartment may underlie the observed association between CD4^+^CD28^null^ T-cell expansion and reduced immune response to heterologous antigens.

## DISCUSSION

In this proof-of-concept study, we demonstrate that subclinical reactivation of CMV drives expansion of the CD4^+^CD28^null^ T-cell subset that has previously been associated with increased risk of infection and mortality in AAV. We show that CMV reactivation and consequent expansion of CD4^+^CD28^null^ T cells are associated with impaired immune response to a T-cell–dependent PCV13, and that antiviral therapy safely suppresses subclinical reactivation of CMV and limits the expansion of CD4^+^CD28^null^ T cells. Moreover, our findings show that reduction of CD4^+^CD28^null^ T cells is associated with improved immune response to pneumococcal vaccination, suggesting that suppression of CMV may have important therapeutic benefit in AAV, in improving the immune response to heterologous antigens. This is of significant clinical relevance, as infection represents a leading cause of death in patients with AAV and the immune response to vaccination is currently suboptimal.

CMV-specific T cells in healthy individuals fluctuate over time, suggesting a degree of ongoing subclinical CMV reactivation [26, 27]. In our study, subclinical CMV reactivation was completely suppressed by valacyclovir. After cessation of treatment, CMV DNA was again detected in the urine of some patients, suggesting that such episodes represent subclinical viral reactivation rather than viral DNA from uroepithelial cells that might have come into contact with latent virus. We found that subclinical CMV reactivation occurred in more than a quarter of AAV patients in remission over 12 months, indicating that CMV reactivation is a frequent event in these patients. It is anticipated that viral reactivation will be even higher during the acute phase of the disease, at a time when patients are exposed to intensive immunosuppressive therapy and heightened systemic inflammation [28]. In keeping with this, we have previously observed that the proportion of CD4^+^CD28^null^ T cells increases during the first year in patients with AAV [4]. Taken together with our current findings, this suggests a heightened degree of subclinical CMV reactivation during this time.

CMV is increasingly recognized as one of the most immunodominant infections encountered by the human immune system. The virus profoundly modulates the immune system with up to 10% of the CD4 and 40% of the CD8 compartments being comprised of CMV-specific T cells [29, 30]. Previous studies in the general population have suggested that this CMV-induced “memory inflation” is associated with suppression of the memory response to heterologous antigens such as Epstein-Barr virus [14] and influenza viruses [31]. Our in vitro studies suggest that expansion of the memory response to CMV, represented by the CD4^+^CD28^null^ T-cell subset, is associated with a progressive decline in the functional capacity of the CD4 compartment. Moreover, the proportion of multifunctional CD4 T cells was found to correlate with the immune response to pneumococcal vaccination. CD4 T-cell help is essential for B-cell clonal expansion and antibody synthesis [32]. Hence, reduced functional capacity of the CD4 compartment is likely to represent one of the mechanisms by which CMV infection leads to the observed impaired immunity to heterologous antigens, particularly in the context of a T-cell–dependent pneumococcal vaccine.

Our study has certain limitations. We did not achieve our recruitment target of 50 patients for the clinical trial, due to fewer patients being eligible for inclusion in the trial than originally anticipated. Nevertheless, valacyclovir treatment completely suppressed subclinical CMV reactivation and reduced CD4^+^CD28^null^ T-cell expansion in treated patients. We assessed CMV reactivation on a monthly basis. It is possible that reactivation episodes occurring in the intervening period may have been missed; however, the likelihood of this would be expected to be equally distributed between the treatment and control groups. There was a nonsignificant difference in baseline CD4^+^CD28^null^ T-cell percentage between the 2 groups. To account for this, paired ratio *t* test analysis of change in CD4^+^CD28^null^ T cells examined differences in the ratios of paired values rather than absolute difference. Due to practical considerations, our study was open-label. However, laboratory staff analyzing samples comprising primary outcome data were blind to treatment allocation.

The results presented here support a mechanism whereby CMV infection reduces the immune response to heterologous antigens in patients with AAV. Our findings suggest that this is mediated by subclinical reactivation of the virus and consequent expansion of the CD4^+^CD28^null^ T-cell subset that is associated with a reduction in the overall functional capacity of the CD4 compartment. Importantly, suppression of CMV reactivation with antiviral therapy limits CD4^+^CD28^null^ T-cell expansion, and this in turn is associated with improved response to a T-cell–dependent pneumococcal vaccine.

Our findings provide proof of principle for the potential benefit of CMV suppression in AAV and support the design of larger studies to determine the frequency of subclinical reactivation of CMV during the more active phase of the disease, and to investigate the potential for CMV suppression to improve the immune response to vaccination and reduce risk of infection, the leading cause of death in AAV.

## Supplementary Data

Supplementary materials are available at *The Journal of Infectious Diseases* online. Consisting of data provided by the authors to benefit the reader, the posted materials are not copyedited and are the sole responsibility of the authors, so questions or comments should be addressed to the corresponding author.

## Supplementary Material

Supplementary Material

## Notes

***Disclaimer.*** The funding sources had no role in the design and conduct of the study; collection, management, analysis, and interpretation of the data; preparation, review, or approval of the manuscript; or decision to submit the manuscript for publication. The views expressed are those of the authors and are not necessarily those of the National Health Service, the National Institute for Health Research (NIHR), or the UK Department of Health.

***Financial support.*** This research was funded by a Wellcome Trust Research Training Fellowship Grant (number 097962/Z/11/Z) and by Vasculitis UK. The study was conducted within the Birmingham NIHR/Wellcome Trust Clinical Research Facility (Birmingham, United Kingdom).

***Potential conflicts of interest.*** All authors: No reported conflicts of interest. All authors have submitted the ICMJE Form for Disclosure of Potential Conflicts of Interest. Conflicts that the editors consider relevant to the content of the manuscript have been disclosed.

Presented in part: Academy of Medical Sciences Meeting, London, United Kindgom, 2017; and American Society of Nephrology Kidney Week Meeting, Chicago, Illinois, 2017.
