# Supplementary material for: Subclinical Reactivation of Cytomegalovirus Drives CD4+CD28null T-Cell Expansion and Impaired Immune Response to Pneumococcal Vaccination in Antineutrophil Cytoplasmic Antibody–Associated Vasculitis
Source: J Infect Dis. 2018 Aug 9;219(2):234–44. doi: 10.1093/infdis/jiy493 (PMC6306020; doi:10.1093/infdis/jiy493)
Supplement: Supplementary Material [file jiy493_suppl_supplementary-material.docx]

**SUPPLEMENTARY MATERIAL**

Table 1 Antibodies used for flow cytometric analysis

| Marker | Fluorochrome | Isotype | Clone | Concentration  μL / test | Manufacturer |
| --- | --- | --- | --- | --- | --- |
| CD3 | Brilliant Violet 650 | Mouse IgG2a, κ | OKT3 | 12 μg/mL  2 μL | Biolegend |
| CD4 | Brilliant Violet 605 | Mouse IgG2b, κ | OKT4 | 100 μg/mL  2 μL | Biolegend |
| CD28 | eFluor 450 | Mouse IgG1, κ | CD28.2 | 25 μg/mL  3 μL | eBioscience |
| CD154 | PE | Mouse IgG1, κ | 24-31 | 100 μg/mL  5 μL | eBioscience |
| IFN-γ | PE-CF594 | Mouse IgG1, κ | B27 | 3 μL | BD |
| TNF-α | Alexa Fluor 700 | Mouse IgG1, κ | MAb11 | 50 μg/mL  3 μL | eBioscience |
| IL-2 | PerCP eFLuor 710 | Mouse IgG2a, κ | MQ1-17H12 | 12 μg/mL  3 μL | eBioscience |

Table 2 Change in markers of inflammation and adverse events

|  | Treatment Group (n=19) | Control Group (n=19) |
| --- | --- | --- |
| Change in markers of inflammation from baseline to month 6^*^ | | |
| hsCRP | +30.4% [-36.8, +169.1]  p=0.451 | -11.3% [-53.5, +69.0]  p=0.700 |
| IFN-γ | -81.7% [-96.7, +1.1]  p=0.051 | +16.6% [-34.3, +107.1]  p=0.580 |
| TNF-α | -38.8% [-65.7, +9.3]  p=0.092 | -14.1% [-49.9, +47.2]  p=0.560 |
| IL-2 | -81.6% [-95.1, -30.7]  p=0.015 | -16.9% [-47.8, +32.3]  p=0.414 |
| IL-6 | -52.2% [-81.3, +22.0]  p=0.115 | -8.6% [-36.4, +31.4]  p=0.610 |
| IL-10 | -31.8% [-65.6, +35.4]  p=0.256 | -18.9% [-45.9, +21.4]  p=0.290 |
| IL-17 | -66.1% [-91.3, +32.7]  p=0.113 | -59.5 [-89.9, +62.2]  p=0.188 |
| Adverse events^†^ | | |
| Hospital admissions, n | 6 | 6 p=1.000 |
| Infections, n | 15 | 16 p=1.000 |
| Gastrointestinal AE, n | 16 | 6 p=0.003 |
| Episodes of acute kidney injury, n | 2 | 1 p=1.000 |
| Episodes of anaemia, n | 2 | 1 p=1.000 |
| Episodes of liver dysfunction, n | 1 | 0 p=1.000 |

^*^Proportionate change with 95% CI (paired ratio t-test)

^†^Adverse events (AE) to end of the study showing number of patients with AE and comparison between treatment and control groups.

hsCRP: highly-sensitive C-reactive protein

**
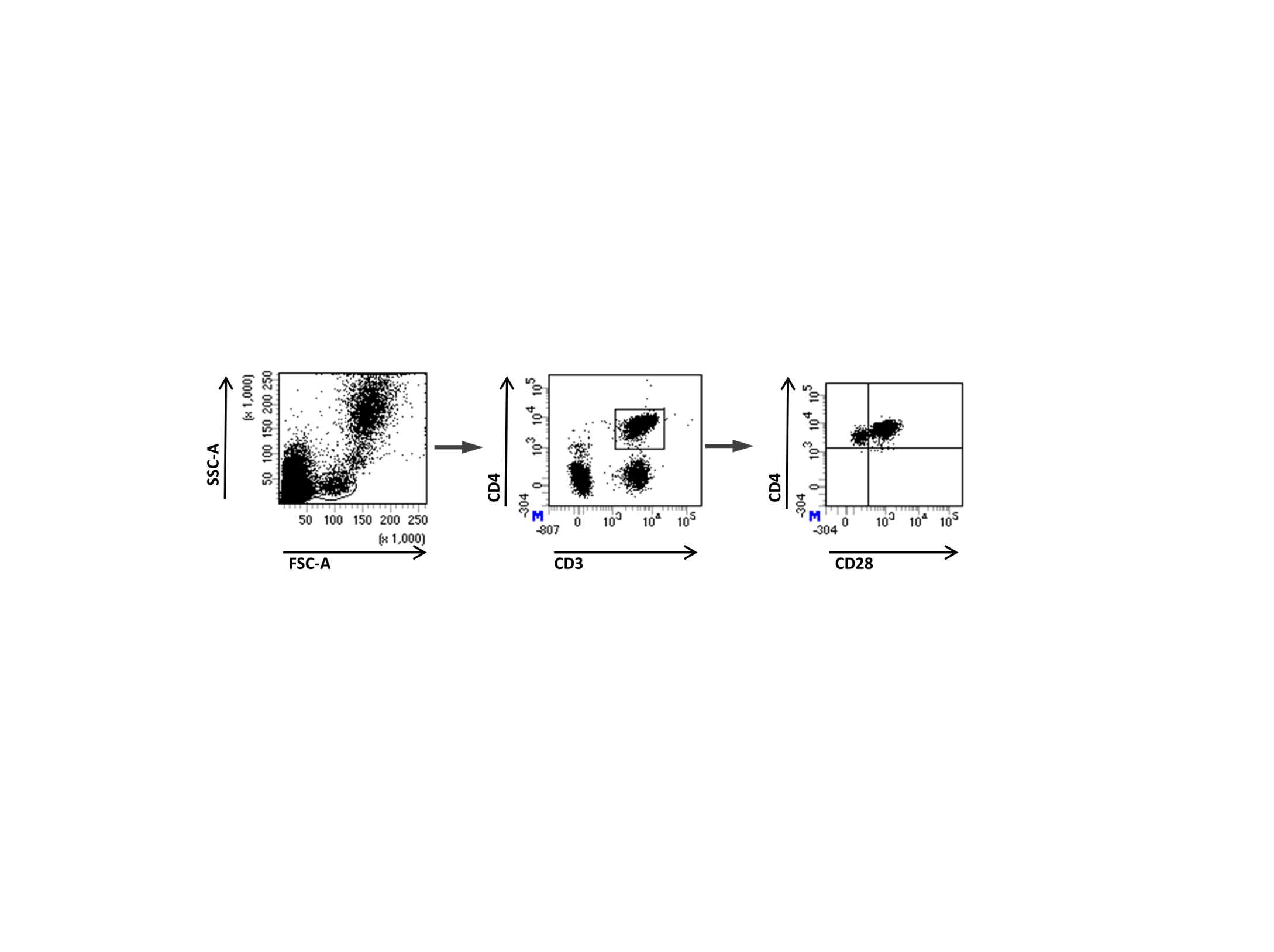
**

**Figure 1 Gating strategy for whole blood staining**

Whole blood was stained with monoclonal antibodies as detailed in the methods section. Sequential gating was performed as follows: lymphocyte gate according to forward scatter (FSC-A) and side scatter (SSC-A) parameters, CD3+CD4+ gate, CD28 gate to identify CD4+CD28null and CD4+CD28+ T-cells.

**
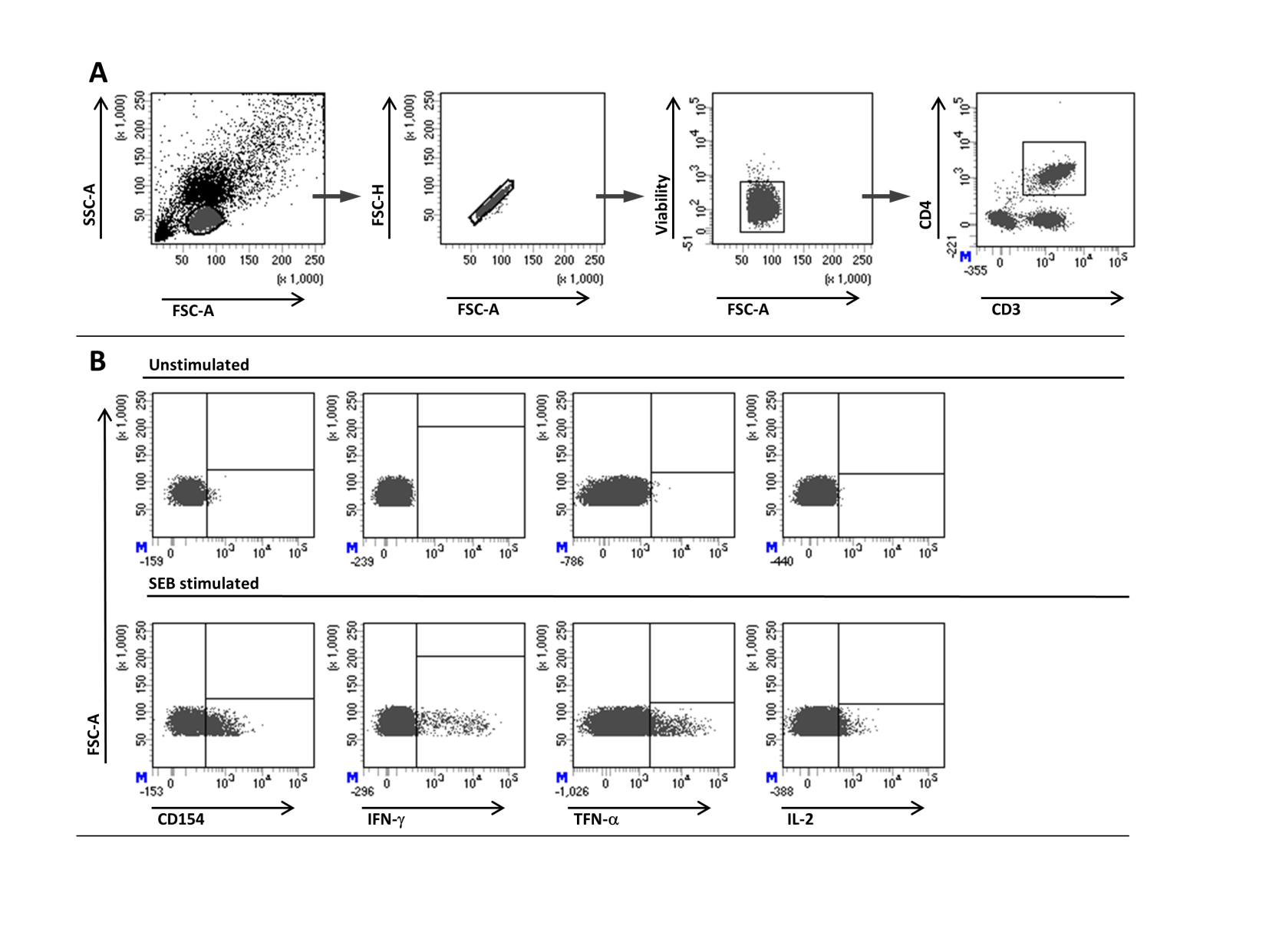
**

**Figure 2 Gating strategy for SEB stimulated PBMC**

PBMC were stimulated with SEB as detailed in the methods section. **A.** Sequential gating was performed as follows: lymphocyte gate according to forward scatter (FSC-A) and side scatter (SSC-A) parameters, single cell gate according to FSC area (FSC-A) and FSC height (FSC-H) parameters, live/dead cell gate, CD3+CD4+ gate **B.** Cytokine and CD154 expression shown for CD3+CD4+ T-cells. Gating for cytokines and CD154 expression was set based on unstimulated cells from the same patient incubated under the same conditions as SEB stimulated cells.

**
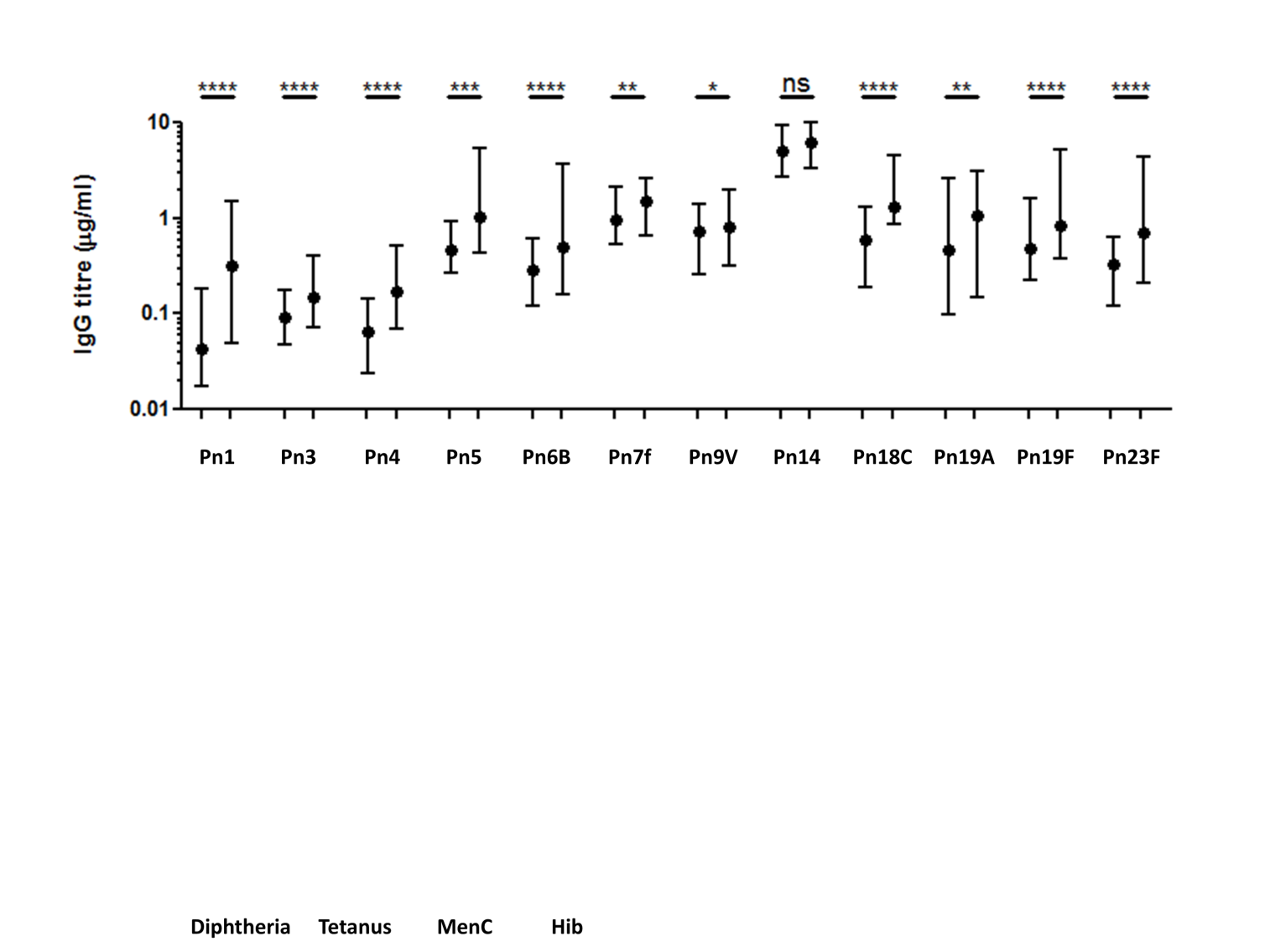
**

**Figure 3 Pre and 4-week post PCV-13 vaccination IgG titre**

Paired pre- and 4 week post-vaccination IgG titre shown for pneumococcal (Pn) serotypes (n=36). Symbols and error bars represent median and interquartile range respectively. IgG titre shown on a logarithmic scale. ****, p ≤ 0.0001; ***, p ≤ 0.001; **, p ≤ 0.01; *, p ≤ 0.05.
